# Purification and Identification of Antioxidant Peptides from Enzymatic Hydrolysates of Tilapia (*Oreochromis niloticus*) Frame Protein

**DOI:** 10.3390/molecules171112836

**Published:** 2012-11-01

**Authors:** Jian Fan, Jintang He, Yongliang Zhuang, Liping Sun

**Affiliations:** College of Chemistry and Engineering, Kunming University of Science and Technology, Kunming 650500, China; Email: fj_3332000@yahoo.com.cn (J.F.); kmustslp@hotmail.com (J.H.); kmylzhuang@yahoo.com.cn (Y.Z.)

**Keywords:** tilapia frame protein, enzymatic hydrolysis, antioxidant peptide, purification, amino acid sequence

## Abstract

Tilapia frame protein was hydrolyzed by different proteases, including properase E, pepsin, trypsin, flavourzyme, neutrase, gc106 and papain, to obtain antioxidant peptides. The tilapia frame protein hydrolysate (TFPH) obtained by trypsin exhibited the highest degree of hydrolysis and antioxidant activity. Three series of peptides (TFPH1, TFPH 2 and TFPH 3) were obtained by ultrafiltration of TFPH through molecular weight cut-off membranes of 5, 3 and 1 kDa, respectively, and their IC_50_ values on scavenging 1,1-diphenyl-2-picrylhydrazyl (DPPH) radical, superoxide anion radical (^•^O_2_), hydrogen peroxides (H_2_O_2_) and hydroxyl radical (•OH) activities were determined and compared with glutathione (GSH). The results showed that TFPH1 had the highest antioxidant activity. TFPH1 was further purified using ion exchange chromatography, gel filtration chromatography, and reversed phase high performance liquid chromatography (RP-HPLC). Finally, two antioxidant peptides were identified and the amino acid sequences were identified as Asp-Cys-Gly-Tyr (456.12 Da) and Asn-Tyr-Asp-Glu-Tyr (702.26 Da), respectively. The IC_50_ values of two peptides on hydroxyl radical scavenging activity were 27.6 and 38.4 μg/mL, respectively.

## 1. Introduction

Reactive oxygen species (ROS) are unavoidable metabolic byproducts of normal aerobic metabolism [1]. ROS can attack macromolecules such as membrane lipids, proteins, and DNA, leading to many health disorders such as hypertension, cardiovascular, cancer, diabetes mellitus, and neurodegenerative and inflammatory diseases with severe tissue injuries [2]. Antioxidants may have a positive effect on human health as they can protect the human body against damage by ROS.

In recent years, peptides from many marine animal sources such as sea urchin gonad [3], smooth hound protein [4], loach protein [5], sardinelle by-products proteins [6], jumbo squid skin [7], grass carp muscle [8], horse mackerel viscera protein [9], sea cucumber [10], alaska pollack skin [11] and pacific hake [12] have been found to possess antioxidant activity. In addition, fish frame proteins such as hoki frame protein [13], alaska pollack frame protein [14] and tuna backbone protein [15] have also proven to be good sources of antioxidant peptides. In those studies, the antioxidant activity was evaluated by scavenging free radicals and ROS *in vitro* assays, such as 1,1-diphenyl-2-picrylhydrazyl (DPPH) radical scavenging, hydroxyl (•OH) radical scavenging, oxygen radical absorbance capacity (ORAC), and superoxide anion radical (^•^O_2_).

Tilapia (*Oreochromis niloticus*) is important species in freshwater aquaculture. It is the third most widely cultured fish, after carp and salmonids [16]. Over the past years, the production of tilapia has been increased steadily and has become one of China’s leading aquatic product exports. The tilapia fish processing industry generates a wide variety of by-products such as heads, skins, viscera and frames. In view of utilizing these fish industry wastes, tilapia by-product proteins and protein hydrolysates are being studied by several researchers all over the World. Jamilah *et al*. [17] isolated gelatin from tilapia skin and studied its properties. Tilapia skin gelatin hydrolysates were proved to be a good antioxidant resource [18], and Dekkers *et al*. [19] showed that tilapia protein hydrolysates in mahi mahi red muscle had good oxidative stability.

However, there are few reports on antioxidant peptides from tilapia frame protein. Fish frame is one of the major fractions of fish wastes (accounting for more than 15% of the fish weight), and it contains around 30% protein [20]. This protein could be a good candidate for nutraceuticals. Therefore, the objective of this study was to prepare tilapia protein hydrolysates using different proteases, evaluate their antioxidant properties using different *in vitro* systems, and further purify antioxidant peptides.

## 2. Results and Discussion

### 2.1. Preparation of Tilapia Frame Protein Hydrolysates (TFPH)

In the present study, tilapia frame protein was separately hydrolyzed by properase E, pepsin, trypsin, flavourzyme, neutrase, gc106 and papain, for the production of antioxidant peptides. As shown in Table 1, the degree of hydrolysis (DH) of the hydrolysates were determined and was observed to be 13.8, 15.1 and 12.7% for properase E, trypsin and flavourzyme, respectively, the other proteolytic enzymes showed DH values lower than 10%.

**Table 1:** The degree of hydrolysis (DH) and antioxidant activities of the hydrolysates from different protease treatments.

Enzyme	DH	Antioxidant activities			
		DPPH	^•^O_2_	H_2_O_2_	•OH
Properase E	13.8 ± 1.2 ^b^	50.8 ± 2.6 ^b^	42.6 ± 3.1 ^b^	70.0 ± 2.7 ^a^	84.5 ± 5.1 ^a^
Pepsin	5.3 ± 0.3 ^d^	26.0 ± 1.5 ^e^	12.9 ± 0.6 ^d^	28.6 ± 0.6 ^e^	23.7 ± 1.5 ^d^
Trypsin	15.1 ± 0.9 ^a^	70.1 ± 4.2 ^a^	58.5 ± 2.8 ^a^	72.2 ± 3.8 ^a^	89.0 ± 4.1 ^a^
Flavourzyme	12.7 ± 0.5 ^b^	54.4 ± 3.5 ^b^	40.4 ± 3.2 ^b^	59.4 ± 4.1 ^b^	68.9 ± 3.1 ^b^
Neutrase	8.6 ± 0.4 ^c^	33.8 ± 1.6 ^d^	24.3 ± 1.8 ^c^	41.5 ± 1.1 ^d^	57.2 ± 2.5 ^c^
GC106	3.8 ± 0.1 ^e^	26.7 ± 0.9 ^e^	11.5 ± 0.7 ^d^	29.1 ± 1.4 ^e^	24.3 ± 0.9 ^d^
Papain	9.5 ± 0.8 ^c^	41.5 ± 2.1 ^c^	26.1 ± 1.6 ^c^	50.7 ± 2.8 ^c^	59.6 ± 1.6 ^c^

Values not sharing a common letter are significantly different at *p* < 0.05.

The antioxidant activity of a substance can be identified by assessing scavenging activities on free radicals generated in oxidative systems. As shown in Table 1, peptides from seven hydrolysates were evaluated for their antioxidant activities using scavenging DPPH, ^•^O_2_, H_2_O_2_ and •OH activity assays. Seven hydrolysates could act as significant scavengers of the four radicals, and the hydrolysates from trypsin showed stronger antioxidant activities. Based on the results, trypsin was chosen to prepare tilapia frame protein hydrolysates (TFPH).

### 2.2. Ultrafiltration

Ultrafiltration is a technique used commonly both on the laboratory and commercial scale to fractionate, purify, and concentrate proteins and peptides [21]. It has been reported to be a useful method for improving the antioxidant activities of protein hydrolysates [13,22,23]. In this study, TFPH was separated into three fractions (TFPH 1 with MW < 1 kDa, TFPH 2 with 1 kDa < MW < 3 kDa and TFPH 3 with 3 kDa < MW < 5 kDa) by ultrafiltration. Based on nitrogen contents, TFPH1, TFPH 2, and TFPH 3 represented 41.9%, 32.7% and 25.4% of hydrolysates, respectively. TFPH1 had the highest yield among all the fractions.

**Figure 1:**
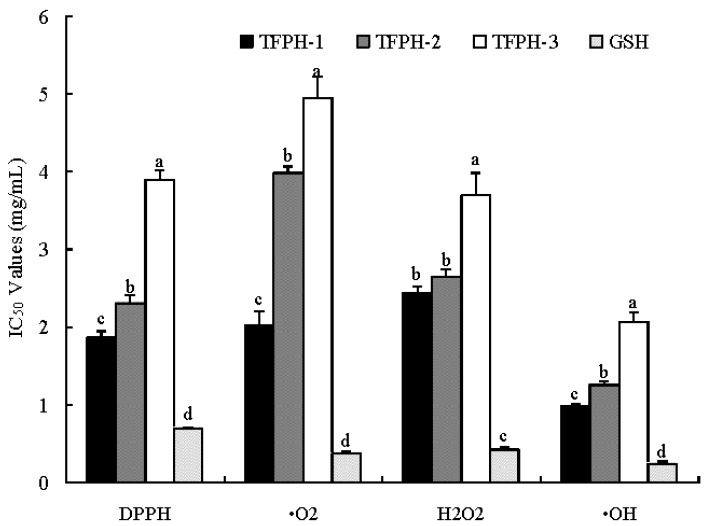
The IC_50_ values of TFPH fractions on scavenging radicals activities. GSH: glutathione. Bars indicate the standard deviation from triplicate determinations. Different letters indicate significant differences (*p* < 0.05).

In order to evaluate the antioxidant activities, the IC_50_ values of three fractions on scavenging activities against the four radicals were calculated and compared with GSH. As shown in Figure 1, the IC_50_ values of TFPH1 and GSH on the DPPH-scavenging activity were 1.92 and 0.69 mg/mL, respectively. TFPH1 had significantly higher DPPH-scavenging activity than other fractions (*p* < 0.05). However, the activity of TFPH1 was lower than that of GSH (*p* < 0.05). You *et al*. [5] reported the IC_50_ value of DPPH-scavenging activity of loach protein hydrolysate was 2.64 mg/mL, which was similar to our result. The ^•^O_2_-scavenging activity of TFPH1 and GSH were 2.01 and 0.37 mg/mL, respectively. The activity of TFPH1 was significant higher than TFPH2 and TFPH3 (*p* < 0.05). Furthermore, TFPH1 also showed high scavenging H_2_O_2_ and •OH activities, and its IC_50_ values were 2.44 and 0.98 mg/mL, respectively, while, the IC_50_ values of GSH were 0.42 and 0.24 mg/mL, respectively. This indicated that TFPH1 might contain peptides which are more easily accessible to the hydroxyl radicals and allows these peptides to trap the radicals more easily [16]. Several studies have looked at the contribution of molecular size of peptide mixtures in protein hydrolysates to their bioactivity. These studies showed that low molecular weight fractions in general contained more potent antioxidative peptides [24,25,26].

### 2.3. Purification of Antioxidant Peptides

Hydroxyl radical is the most reactive radical and can be formed from superoxide anion and hydrogen peroxide in the presence of metal ions, such as copper or iron. The hydroxyl radical has been demonstrated to be highly damaging species in free radical pathology, attacking almost every molecule in living cells. Therefore, the removal of hydroxyl radical is probably one of the most effective defence of a living body against various diseases. Base on this, the scavenging •OH activity was selected as the indicator of purification of antioxidant peptides in the study [16].

#### 2.3.1. Purification of Antioxidant Peptide Using Ion-Exchange Chromatography

**Figure 2:**
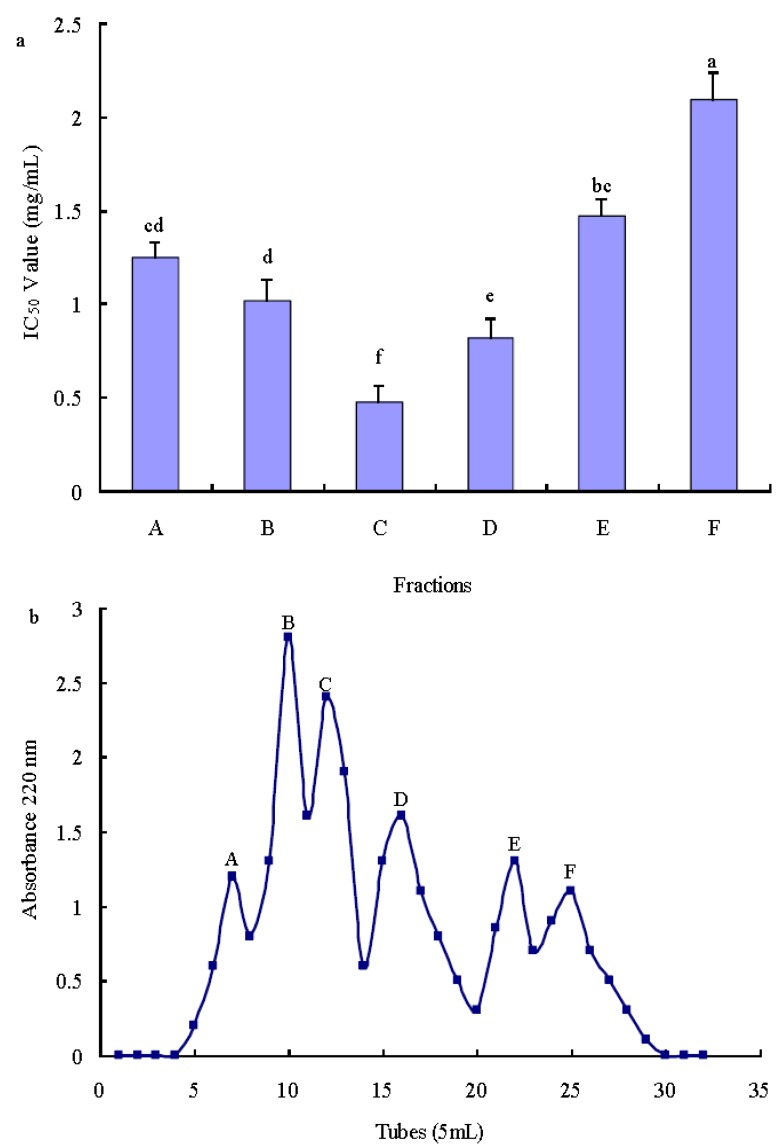
TFPH1 was separated by SP Sephadex C-25 gel chromatography (**a**), and the IC_50_ value (mg/mL) of each fraction was measured by hydroxyl radical scavenging activity (**b**). Different letters indicate significant differences (*p* < 0.05)

SP Sephadex C-25 (main functional group: sulphopropyl) was one of the strong cation exchangers and it was widely utilized for separating antioxidant peptides [16,24]. In order to prepare the antioxidant peptides, TFPH1 was separated by SP-Sephadex C-25 ion exchange chromatography, showing six different peaks, named A, B, C, D, E and F (Figure 2a). As shown in Figure 2b, fraction C was found to possess the strongest hydroxyl radical scavenging activity, among six fractions, with the IC_50_ value of 0.47 mg/mL.

#### 2.3.2. Purification of Antioxidant Peptide Using Gel Filtration

**Figure 3:**
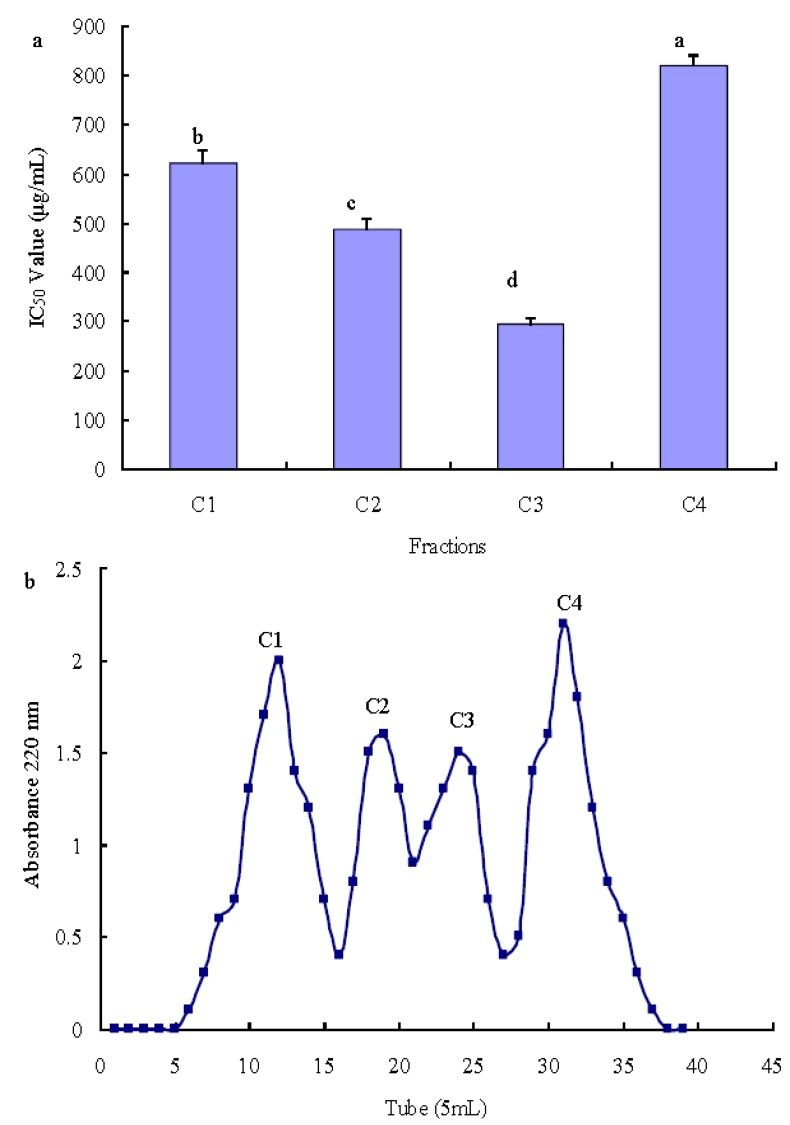
Sephadex G-25 gel chromatography of Fraction C (**a**) and the IC_50_ value (μg/mL) of each fraction was measured by hydroxyl radical scavenging activity (**b**). Different letters indicate significant differences (*p* < 0.05).

Peptide length was considered to be closely related to biological activities. Earlier studies have shown that peptide length had a significant effect on antioxidant activity [25,26], so fraction C was further fractionated by gel filtration chromatography on a Sephadex G-25 column, showing four different peaks, named C1, C2, C3, and C4 (Figure 3a). The C3 fraction exhibited stronger antioxidant activity than the other fractions (*p* < 0.05), with the IC_50_ value being 280.97 μg/mL (Figure 3b).

#### 2.3.3. Purification of Antioxidant Peptide Using RP-HPLC

**Figure 4:**
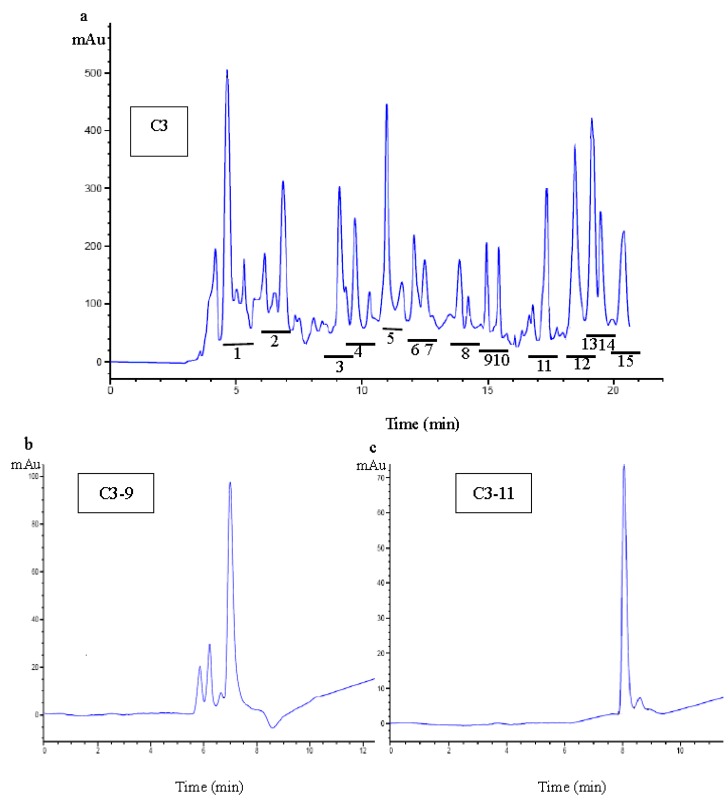
Chromatography of Fraction C3 was separated by semi-preparative RP-HPLC (**a**), and numbers 1–15 represented the elution peaks of C3-1–C3-15. Chromatography of C3-9 (**b**) and C3-11(**c**) were further separated by RP-HPLC analytical column.

The C3 fraction was further isolated by RP-HPLC on the semi-preparative C_18_ column using a linear gradient of acetonitrile containing 0.1% TFA. As shown in Figure 4a, fifteen peaks, designated as C3-1 to C3-15, were collected separately. Each fraction was pooled and concentrated and the C3-9 and C3-11 showed the higher scavenging •OH activity at a concentration of 50 μg/mL. Fractions C3-9 and C3-11 were further identified by RP-HPLC on the Zorbax SB-C_18_ analysis using a linear gradient of acetonitrile containing 0.1% TFA. One main peak was obtained from C3-9 (Figure 4b) and C3-11 (Figure 4c), with the IC_50_ values being 27.6 and 38.4 μg/mL, respectively.

### 2.4. Identification of Amino Acid Sequence of Antioxidant Peptide

**Figure 5:**
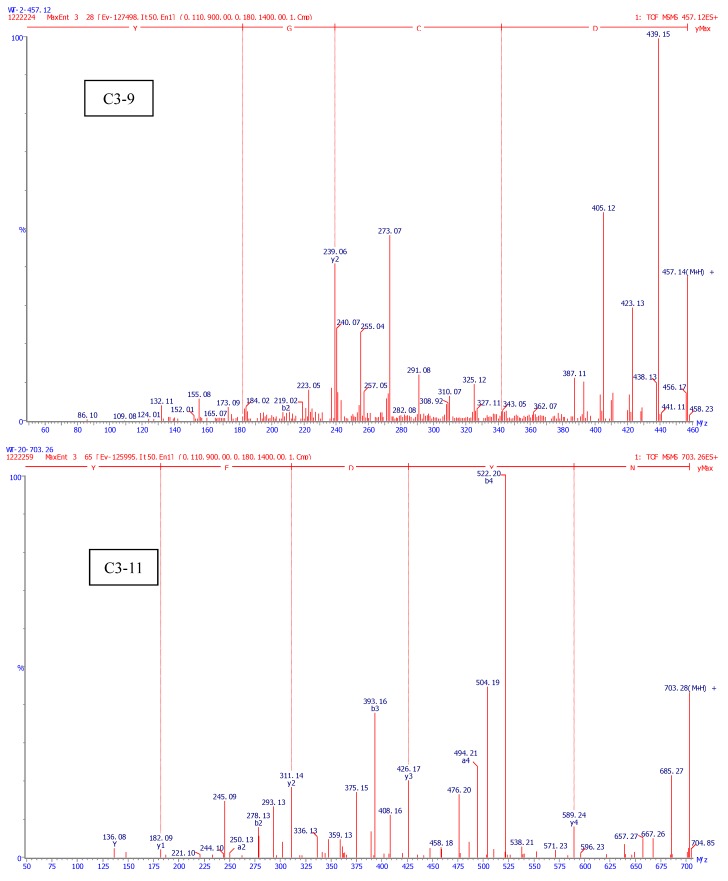
Identification of molecular mass and amino acid sequences of the purified peptides (C3-9 and C3-11) by Nano-LC-ESI-Q-TOF MS/MS.

As shown in Figure 5, the two peptides (C3-9 and C3-11) were obtained from TPFH and the molecular weights were 456.12 and 702.26 Da, respectively. The amino acid sequences were Asp-Cys-Gly-Tyr and Asn-Tyr-Asp-Glu-Tyr, respectively.

In the sequences of our purified peptides, direct proton-donators such as Tyr, Cys, Gly, Glu and Asp are present, which is very important to the radical-scavenging activity of peptides due to their ability to quench unpaired electrons or radicals by donating protons [27].

Some studies have reported that peptide sequences containing Tyr showed strong antioxidant activity. Especially, when the presence of Tyr was at terminals of the peptide sequence, such as Leu-Pro-His-Ser-Gly-Tyr, Leu-His-Tyr, Val-Thr-Pro-Tyr and Asp-Val-Cys-Gly-Arg-Asp-Val-Asn-Gly-Tyr. The antioxidant activity of Tyr may be explained by the special capability of phenolic groups to serve as hydrogen donors, which is one mechanism of inhibiting the radical-mediated peroxidizing chain reaction [28,29]. Our results were similar to these previous studies, and the two peptides obtained from TPFH contained Tyr at the terminus.

Previous studies show Cys is hydrophobic in nature and can interact directly with free radicals by donating the sulfur hydrogen [9], so the presence of Cys is one of the reasons for the good antioxidant activity of the isolated peptide. Moreover, hydroxyl radicals could be formed from superoxide anion and hydrogen peroxide in the presence of transition metal ions, such as Fe^2+^ and Cu^2+^, so chelating metal ions may inhibit the formation of the hydroxyl radical [16]. Acidic amino acids such as Asn and Glu play important roles in the chelation of metal ions by their side chains [30,31]. In addition, Rajapakse *et al*. [32] reported that presence of Asp seemed to play a vital role irrespective of its position as observed in several antioxidative peptide sequences. It was assumed that the presence of both Cys and Asp residues within the sequence of C3-9, and Asn, Asp and Glu within the sequences of C3-11 seemed to play a vital role for their antioxidant activities.

## 3. Expermental

### 3.1. Materials

The tilapia frame was provided by New Ocean Food Co., Ltd. (Kunming, China). Neutrase, gc106, trypsin, flavourzyme and properase E were purchased from Genencor International Co. (Wuxi, China). Papain, pepsin and 1, 1-diphenyl-2-pycrylhydrazyl (DPPH) were purchased from Sigma Chemical Co., (St. Louis, MO, USA). Sephadex G-25 and SP Sephadex C-25 were purchased from GE Healthcare (Fairfield, CT, USA). Acetonitrile (HPLC grade) was purchased from Merck KGaA (Darmstadt, Germany). All other reagents used in this study were analytical grade.

### 3.2. Enzymatic Hydrolysis

Tilapia frame was treated with high-pressure cooking at 120 °C for 30 min to soften the backbone and then homogenized in a high speed tissue homogenizer (Cany Precision Instrument Co., Ltd., Shanghai, China).

The tilapia frame protein was hydrolyzed by seven enzymes, respectively. Tilapia frame homogenate (protein concentration 25 mg/mL) was adjusted to optimum (based on the manufacturer recommendations) of the respective enzyme used (Table 2). The enzymatic hydrolysis process was without pH control and the enzyme inactivation was accomplished by heating for 3 min in boiling water. The obtained hydrolysates were centrifuged at 8,000 × *g* for 10 min and the supernatant was collected.

**Table 2:** The optimum conditions (based on the manufacturer recommendations) for seven proteases hydrolyzing tilapia frame protein.

Enzyme	Activity (U/g)	Source	Buffer	pH	T (°C)	Time (h)	[E]/[S] (g/g)
Properase E	6.5 × 10^4^	Bacillus	0.05 M Na_2_HPO_4_-NaH_2_PO_4_	9.0	50	4	1:50
Pepsin	4.5 × 10^4^	Porcine stomach	0.05 M Glycine-HCl	2.0	37	6	1:50
Trypsin	9.5 × 10^4^	Bovine pancreas	0.05 M Na_2_HPO_4_-NaH_2_PO_4_	7.5	45	3	1:100
Flavorzyme	5.5 × 10^4^	Aspergillus	0.05 M Na_2_HPO_4_-NaH_2_PO_4_	7.0	45	4	1:100
Neutrase	2.0 × 10^5^	Bacillus	0.05 M Na_2_HPO_4_-NaH_2_PO_4_	7.0	45	4	1:50
GC106	5.0 × 10^4^	Aspergillus	0.05 M Na_2_HPO_4_-NaH_2_PO_4_	4.5	45	6	1:33
Papain	8.5 × 10^4^	Papaya latex	0.05 M Na_2_HPO_4_-NaH_2_PO_4_	6.0	37	3	1:100

### 3.3. Determination of the Degree of Hydrolysis

Degree of hydrolysis (DH) was evaluated according to ninhydrin colorimetric method based on the equation: DH (%) = h (mmol/g)/h_tot_ (mmol/g) × 100%, where h is the number of broken peptide bonds per unit weight; and h_tot_ is the total number of peptide bonds per unit weight. The h_tot_ for tilapia frame protein was 9.3 mmol per gram protein [33,34].

### 3.4. Antioxidant Activities Assay

#### 3.4.1. 1,1-Diphenyl-2-picrylhydrazyl (DPPH) Radical Scavenging Activity Assay

The DPPH radical-scavenging activity was measured according to the method of Huang and Mau [35]. An aliquot of 1 mL of hydrolysates was mixed with 1 mL of methanol solution containing 1 mM DPPH radicals. The mixture was allowed to stand for 40 min in the dark, and the absorbance was monitored at 517 nm. Distilled water was used instead of hydrolysates as a blank. GSH was used as a positive control. Scavenging DPPH activity was calculated according to the following equation [Equation (1)]:

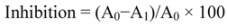
(1)
where A_1_ is sample absorbance and A_0_ is blank absorbance.

#### 3.4.2. Superoxide Anion (^•^O_2_) Scavenging Assay

^•^O_2_ scavenging activity was assessed by chemiluminescence analysis of a pyrogallol luminol system with a slight modification [36]. A volume of 50 μL of freshly prepared pyrogallol solution (0.625 mM), 100 μL of hydrolysates and 850 μL of luminal solutions (1 mM, in 0.1 M NaCO_3_) were mixed in a reaction tube. Luminol chemiluminescence of the system was measured using an ultraweak luminescence analyzer (BPCL, Beijing, China). Distilled water was used instead of hydrolysates as a blank. GSH was used as a positive control. Scavenging ^•^O_2_ activity was calculated according to Equation (2):

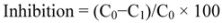
(2)
where C_1_ is sample chemiluminescence and C_0_ is blank chemiluminescence.

#### 3.4.3. Hydrogen Peroxide (H_2_O_2_) Scavenging Assay

H_2_O_2 _scavenging activity of hydrolysates was determined according to the method described by Zhuang *et al*. [37] with a few modifications. A solution of hydrogen peroxide (4 mM) was prepared in phosphate buffered saline (0.1 M, pH 7.4). Volumes of 200 μL of samples in distilled water were mixed with 2.8 mL of hydrogen peroxide solution. Absorbance of the mixture was measured at 230 nm after 10 min against the blank solution in phosphate buffer without hydrogen peroxide. Distilled water was used instead of hydrolysates as control. GSH was used as a positive control. The percentage of H_2_O_2_ scavenging of hydrolysates was calculated according to the Equation 1.

#### 3.4.4. Hydroxyl Radical (•OH) Scavenging Assay

•OH scavenging activity was assessed using an ascorbic acid-Cu^2+^-hydrogen superoxide-yeast suspension system with a slight modification [38]. A volume of 200 μL of freshly prepared ascorbic acid solution (2 mM), 0.4 mL of CuSO_4_ (2 mM), 50 μL of luminal (1 mM, in 0.1 M NaCO_3_), 200 μL of yeast suspension (7.5 g per 100 mL) and 600 μL of hydrolysates were injected into the reaction tube and kept in a water bath at 37 °C for 30 min, Then 600 μL of H_2_O_2_ solution (6.8 mM) were added to start the reaction. Luminol chemiluminescence of the system was measured using an ultraweak luminescence analyzer. Distilled water was used instead of hydrolysates as a blank. GSH was used as a positive control. •OH scavenging activity was calculated according to Equation (2).

### 3.5. Purification of Antioxidant Peptides

#### 3.5.1. Ultrafiltration

The supernatant of hydrolysates showing the strongest antioxidant activity were fractioned using three different molecular weight cut-off (MWCO, 5, 3, 1 kDa) membranes (Yadong Hitech Co. Ltd, Shanghai, China). The fractioned temperature was kept at 4 °C and the pressure was adjusted to 0.15 MPa. Three series of peptides were obtained, noting TFPH 1 with MW < 1 kDa, TFPH 2 with 1 kDa < MW < 3 kDa and TFPH 3 with 3 kDa < MW < 5 kDa. The peptides were lyophilized and stored at −20 °C until future use. Hydrolysate nitrogen contained all nitrogens derived from proteins, peptides, and free amino acids. Protein content was measured according to the Kjeldahl procedure and multiplying the nitrogen value by 6.25.

#### 3.5.2. Ion-Exchange Chromatography

TFPH was loaded onto a cationic exchange column (2.6 × 50 cm) with a SP Sephadex C-25 equilibrated with sodium acetate buffer (20 mM, pH 4.0). The column was washed with the same buffer and eluted with a linear gradient of NaCl concentrations from 0 to 1.0 M at a flow rate of 0.5 mL/min and monitored at 220 nm. Every 5 mL of eluted solution was collected. Fractions with the desire peak were pooled and lyophilized.

#### 3.5.3. Gel filtration Chromatography

The fraction with the highest hydroxyl radical scavenging activity obtained from SP Sephadex C-25 was fractionated through Sephadex G-25 gel filtration column (2.0 × 100 cm) using distilled water as the eluting solvent at 0.5 mL/min flow rate and monitored at 220 nm. Every 5 mL of eluted solution was collected. Fractions with the desire peaks were pooled and lyophilized.

#### 3.5.4. High-Performance Liquid Chromatography (HPLC)

The fraction with the highest hydroxyl radical scavenging activity obtained from Sepahadex G-25 was further separated by reversed phase high performance liquid chromatography (RP-HPLC) on a Zorbax semi-preparative C18 (9.4 × 250 mm) column (Agilent Technologies, Palo Alto, CA, USA), using a linear gradient of acetonitrile containing 0.1% TFA (5–30%, in 30 min) at a flow rate of 2.0 mL/min. The fractions showing the high antioxidant activity were identified on a Zorbax analytical C18 (4.6 × 250 mm) column (Agilent Technologies) at a flow rate of 1.0 mL/min with a linear gradient of acetonitrile containing 0.1% TFA (5–25%, in 20 min). The purification procedures were repeated until enough samples were collected for the activity assay and sequence identification.

#### 3.5.5. Amino Acid Sequence of the Purified Peptides

An accurate molecular mass and amino acid sequence of the purified peptides were determined using a Q-TOF mass spectrometer (Micromass, Altrincham, UK) coupled with an electrospray ionization (ESI) source.

### 3.6. Statistical Analysis

All results were expressed as means ± standard deviation and analyzed by the SPSS 11.5 statistical software. Data were analyzed using one-way analysis of variance (ANOVA). *p* < 0.05 indicated statistical significance.

## 4. Conclusions

Seven proteases were selected to hydrolyze tilapia frame protein. The tilapia frame protein hydrolysate (TFPH) obtained by trypsin showed the highest degree of hydrolysis and antioxidant activity. TFPH was ultrafiltered through the different MWCO membranes and three series of peptides, TFPH 1 with MW < 1 kDa, TFPH 2 with 1 kDa < MW < 3 kDa and TFPH 3 with 3 kDa < MW < 5 kDa, were obtained, respectively. TFPH1 had higher radicals-scavenging activity than TFPH2 and TFPH3 with the highest yield, so TFPH 1 was further fractionated using ion exchange chromatography, gel chromatography and RP-HPLC. Two antioxidant peptides were purified, and their sequences were identified as Asp-Cys-Gly-Tyr (456.12 Da) and Asn-Tyr-Asp-Glu-Tyr (702.26 Da). They displayed high hydroxyl radical scavenging activity with the IC_50_ values of 27.6 and 38.4 μg/mL. Based on these results, these two peptides have the potential to be developed into new health foods.
